# Review of the Free Research Software for Computer-Assisted Interventions

**DOI:** 10.1007/s10278-023-00912-y

**Published:** 2024-01-29

**Authors:** Zaiba Amla, Parminder Singh Khehra, Ashley Mathialagan, Elodie Lugez

**Affiliations:** Toronto Metropolitan University, Toronto, ON Canada

**Keywords:** Research software, Evaluation, Comparison, Segmentation software, Registration software

## Abstract

Research software is continuously developed to facilitate progress and innovation in the medical field. Over time, numerous research software programs have been created, making it challenging to keep abreast of what is available. This work aims to evaluate the most frequently utilized software by the computer-assisted intervention (CAI) research community. The software assessments encompass a range of criteria, including load time, stress load, multi-tasking, extensibility and range of functionalities, user-friendliness, documentation, and technical support. A total of eight software programs were selected: 3D Slicer, Elastix, ITK-SNAP, MedInria, MeVisLab, MIPAV, and Seg3D. While none of the software was found to be perfect on all evaluation criteria, 3D Slicer and ITK-SNAP emerged with the highest rankings overall. These two software programs could frequently complement each other, as 3D Slicer has a broad and customizable range of features, while ITK-SNAP excels at performing fundamental tasks in an efficient manner. Nonetheless, each software had distinctive features that may better fit the requirements of certain research projects. This review provides valuable information to CAI researchers seeking the best-suited software to support their projects. The evaluation also offers insights for the software development teams, as it highlights areas where the software can be improved.

## Introduction

The development of research software in Computer-Assisted Interventions (CAI) and medical image analysis lays the groundwork for numerous achievements in the discipline. This paper presents a comprehensive review of several free software available to CAI investigators. More specifically, ‘computer-assisted interventions’ refers to the use of cutting-edge computer technologies to facilitate the planning and execution of medical interventions, such as radiotherapy, neurosurgery, and orthopedics. In many of these interventions, medical images play a critical role. They provide the necessary information to plan effective interventions, provide intra-operative guidance, and assess the efficacy of the procedure post-intervention. While it is not necessary for relevant software to provide image processing functionalities, many of them do offer such capabilities.

As industrial and academic research groups regularly embark on ambitious biomedical R &D projects, numerous software has been developed to support their efforts. Fortunately, some of them are freely distributed to the research community [1–19]. Due to the lack of standardization and comparability among these software solutions, researchers often struggle to identify the most suitable software for their needs.

Various levels of research software resources are available to the CAI community. These can be classified as follows:

- Software—which is a compilation of computer programs that provide functionalities to a computer system in order to solve specific problems.
- Tools—which are simpler software as they enable a limited set of specific functionalities, such as image viewers providing only basic functionalities such as image annotations.
- Software frameworks and toolkits—which provide a suite of pre-written software components for building software applications.

This study is concerned with reviewing software only, although the boundaries that demarcate the different categories are not always clear. For instance, some software frameworks will also be considered “software.”

Numerous papers have been written to detail either the development of specific software or an extension to an existing one [4–7, 9, 10, 15, 20–22]. These papers typically provide information such as a technical overview of the software’s construction, instructions for its usage, as well as reports on validation studies. On certain occasions, the software development team may compare their software with other similar software that already exists. However, these comparisons could be biased.

A variety of high-level reviews have been conducted on software resources available to the CAI community [23–26]. For instance, Takacs et al. [23] provide a high-level overview of a mix of open-source software toolkits, frameworks, and hardware that support Surgical Robotics research. Similarly, Wolf et al.[24] provide an overview of toolkits and software that support the development of biomedical image processing software. There are also several reviews of medical image viewers [27–29].

The most pertinent software review was conducted by Virzi et al. [26] However, the software was assessed with a particular focus on generating patient-specific 3D models for pelvic surgery planning. To the best of our knowledge, there are currently no in-depth reviews of existing software developed to support CAI research.

Hence, this paper attempts to provide an in-depth analysis of some of the popular and free software for CAI research. Software programs are evaluated based on various criteria, including stress load, multi-tasking, effectiveness of the functionalities, documentation, and support. Indeed, different researchers may prioritize different aspects of the software [22]. We believe that this study can be a valuable resource for research teams when selecting software to support their research projects, as well as those who are developing new extensions to existing software. Additionally, this review can offer software development teams insights into areas of their software that require improvement.

The paper is structured as follows: In the “Methods” section, the software selection criteria and evaluation criteria are presented. The “Results” section presents the comparative ranking results. Additionally, a more detailed review of the individual software assessments is subsequently provided. The “Discussion” section delves into a discussion of the results, provides additional context for their interpretation, and addresses the limitations of this review.

## Methods

### Software Selection Criteria

A set of criteria, used to select software to review, was defined by a diverse team consisting of computer scientists and biomedical researchers, by combining their relevant prior industrial and/or academic experience with selection criteria found in the relevant literature [22, 26, 30]. Specifically, the software should:

- Be free of charge, even if it is not open-source.
- Be easily downloadable, without requiring administrative approval or other similar contingencies. As such, the software should be available online.
- Be easy to install, without requiring extensive technical knowledge or assistance.
- Be a stand-alone software: it should not require additional components.
- Be maintained: a new version should have been released within the past 3 years (for example to fix bugs or add new functionalities).
- Be adopted in a wide range of studies (over a thousand research papers published on Google Scholar should report having used the software).
- Be cross-platform to reach a wider audience and to enable collaboration among research partners.
- Be intended to support CAI research initiatives.
- Support advanced functionalities (for example, a simple image viewer that provides annotation functionalities will not be reviewed).
- Usable by end-users with basic or no programming experience.

Software programs not achieving all of the above-mentioned list of requirements were not selected. The list of selected software includes: 3D Slicer, Elastix, ITK-SNAP, MedInria, MeVisLab, MIPAV, the MITK Workbench, Seg3D.

The software programs were identified based on previous knowledge and mainly from the results of the literature review. Additionally, to ensure thoroughness, a search for software was conducted on Google Scholar using various combinations of the keywords “segmentation software,” “registration software,” and “image analysis.”

### Evaluation Criteria

The below set of criteria was used to compare the software programs with each other:

#### Load Time

This quantitative test evaluated the duration it took for the software to become operational upon launch. Specifically, we examined the time required for the software to be ready for use solely during the initial execution. This is because some applications cache their data in the main memory, which means that subsequent executions do not need to load all the libraries.

#### Stress Load

This quantitative test involves evaluating the software’s performance when subjected to high volumes of requests and data, such as loading multiple thousands of 2D medical images. Indeed, the stress load test is critical for projects that rely on a large number of files. Because certain software did not allow the simultaneous loading of multiple files, a synthesized large image—approximately 4.25GB, created by combining 54,020 2D magnetic resonance images—was used.

#### Multi-tasking

**Fig. 1:**
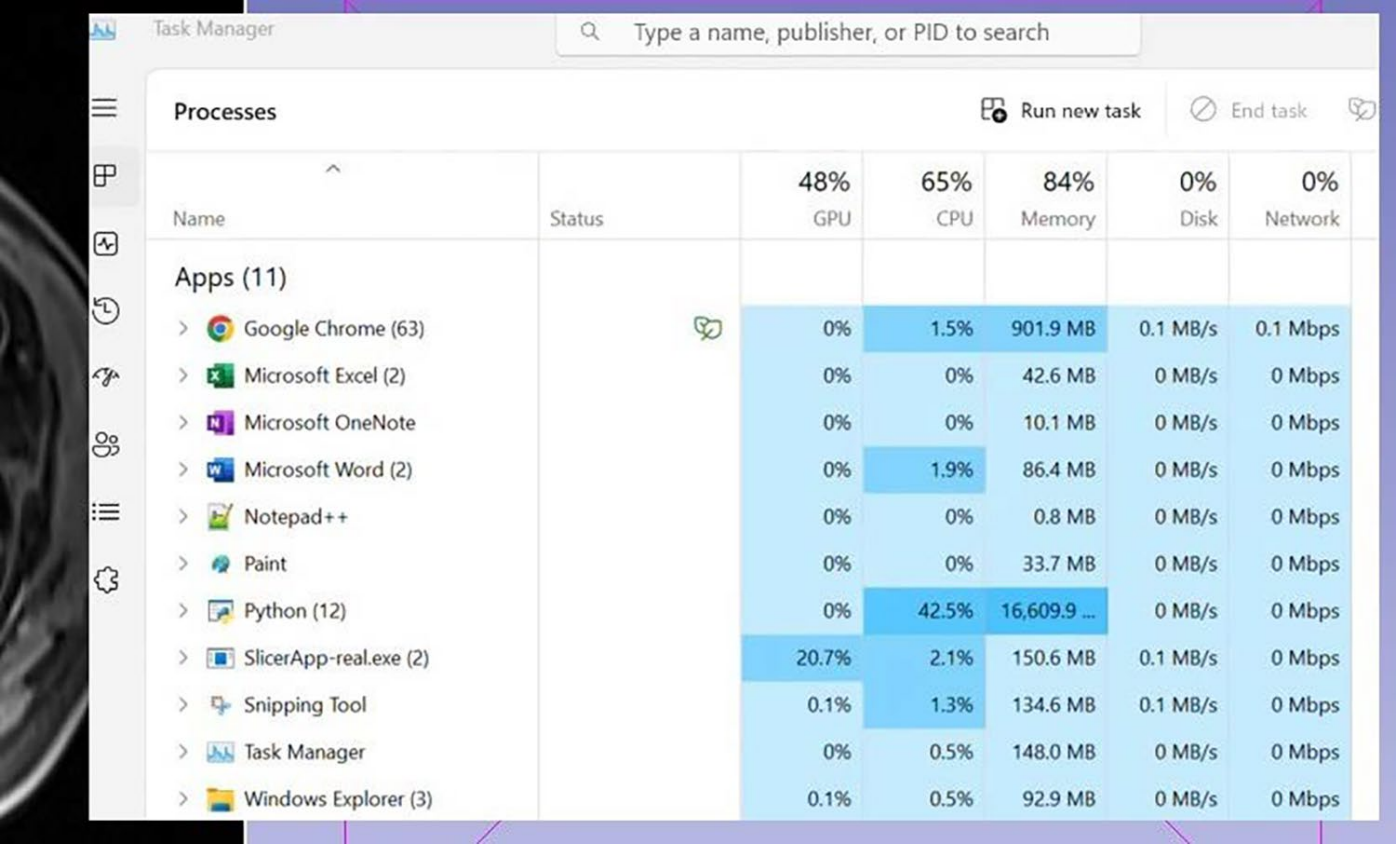
Stress load and multi-tasking tests: the task manager is used to monitor CPU, GPU, and RAM usage

This quantitative test evaluated the software’s performance while other software or processes are running simultaneously. This involved opening several applications simultaneously, such as Word, Excel, over 20 Google Chrome tabs, and running a complex Python script in the background. During this test, the RAM usage was monitored using the task manager (Fig. 1) while conducting image registration between two 3D magnetic resonance images. The objective was to determine if the software excessively consumed resources. Indeed, this can create resource conflicts with other applications that run in the background. In such cases, the software would be deemed to be poor at multi-tasking.

#### Range of Functionalities

This evaluation documented the diversity of the software’s functionalities. This also entailed exploring the completeness of the image segmentation and registration functionalities offered.

#### Effectiveness of Functionalities

This evaluation utilized the software’s image registration and/or segmentation functionalities. The segmentation’s functionality was investigated by manually and (semi-)automatically segmenting a liver, when possible, from a 3D magnetic resonance image. In addition, the prostate was segmented from a 2D transrectal ultrasound image. The registration functionality was tested by aligning two 3D magnetic resonance images together, as well as two 2D ultrasound images together. Through these assessments, the software’s usability, reliability, and the extent of manual intervention required from the user were gauged.

#### Extensibility of the Functionalities

This evaluation examined whether the software allows users to integrate custom functionality into the software. A modular software would enable users to enhance its capabilities through add-ons without modifying the core codebase directly.

#### User-Friendliness

The team analyzed the interactions with the software while performing simple tasks, such as importing images, segmenting images, and registering images. The ranking of software programs was determined by users’ overall satisfaction.

#### Documentation

The team researched the availability of a user guide, tutorials, and other forms of documentation that detail the essential resources for understanding and navigating the software.

#### Technical Support

The team investigated whether users can seek help and support through emails and/or forums.

The quantitative tests (load time, stress load, multi-tasking) provided in-depth details about the software. These performance tests were all conducted on a Surface Laptop Studio, running on Microsoft Windows 11 OS, with an 11th Gen Intel core i7-11,370 H @3.30GHz CPU, 8 cores, 32 GB RAM, and an NVIDIA RTX 3050 GPU. Multiple runs of each of these tests were conducted to eliminate one-time anomalies. A second tester repeated such tests to ensure the results are consistent, independently of computer specifications. The other investigations (namely the range of functionalities, effectiveness of functionalities, extensibility of the functionalities, user-friendliness, documentation, and technical support) were independently performed by three team members, and the resulting rankings were combined. In any case, whenever there was a tie between two software, the best score was given to the software that behaved the most consistently with regard to that criterion.

The software programs were ranked using a scale from 1 to 8 along each evaluation criterion. A rank of 1 would be attributed to the software that performed the best, and 8 was given to the one that performed the worst. In case a software program did not succeed at a test, a “failed” indication was recorded, while if a test could not be applied to a software, a “not applicable” note was reported.

## Results

### Rankings

Table 1 presents the rating results. Detailed insights follow.

**Table 1:** Software rankings based on proposed criteria: 1 for best, 8 for worst

	3D Slicer	Elastix	ITK-SNAP	MedInria	MeVisLab	MIPAV	MITK Workbench	Seg3D
Load times	7	NA	1	5	6	3	4	2
Stress load	3	NA	1	4	failed	failed	2	failed
Multi-tasking	5	1	2	6	3	4	7	NA
Range of functionalities	1	8	6	5	3	2	4	7
Extensibility	Yes	No	No	No	Yes	Yes	Yes	No
Effectiveness of the functionalities								
- Image registration	2	1	3	5	7	4	6	8
- Image segmentation	2	NA	4	7	6	5	1	3
User-friendliness	4	5	1	2	8	6	3	7
Documentation	1	4	3	5	6	8	2	7
Technical support	1	4	2	6	5	8	3	7

“failed” indicates software inability to load the large image

“NA” denotes tests that could not be applied (Elastix lacked UI and cannot segment images; Seg3D’s multi-tasking performance was not compared due to registration pre-processing requirements)

#### Load Times

ITK-SNAP demonstrated the fastest loading time, taking an average of 1.33 s. Seg3D followed with a load time of 4.00 s, while MIPAV took 10.33 s to load. The MITK Workbench required 11.00 s, MedInria took 13.00 s, MeVisLab took 16.67 s, and 3D Slicer had the longest load time at 19.67 s.

#### Stress Load

ITK-SNAP again secured the top position, utilizing only 0.5% of the system’s GPU, 24% of the CPU, and 3000 MB of RAM. The second place was taken by the MITK Workbench, which had higher RAM usage (4840 MB), along with 0.3% GPU utilization and 10% of the system’s CPU. 3D Slicer ranked third, with a similar RAM usage to the MITK Workbench (4590 MB), but utilizing 0% of the GPU and 30% of the system’s CPU. MedInria performed with 0% GPU usage, 55% CPU usage, and 12,700 MB of RAM. MeVisLab, MIPAV, and Seg3D systematically crashed during the stress test.

#### Multi-tasking

Elastix emerged as the top-performing software, utilizing 4% of the system’s GPU, 40% of the CPU, and a minimal 300 MB RAM. ITK-SNAP secured the second position, with 6% GPU usage, a low 20% CPU usage, and 470 MB RAM consumption. MeVisLab ranked third, displaying no GPU usage, but utilizing 50% of the system’s CPU and 514 MB RAM. MIPAV also did not rely on GPU processing, using 49% of the system’s CPU and 650 MB of RAM. 3D Slicer utilized 5% of the system’s GPU, 56% of the CPU, and 849 MB RAM. MedInria, like 3D Slicer, used 5% of the system’s GPU, had a lower CPU usage of 50%, but consumed 1177 MB RAM. Finally, the MITK Workbench utilized 5% of the system’s GPU, exhibited the highest CPU usage at 79%, and consumed 923 MB of RAM.

#### Range of Functionalities

3D Slicer ranked first, as it supports an extensive number of file types. It further boasts a large collection of functionalities made possible through its numerous extensions, totaling 169 at the time of writing. While it is renowned for medical image processing, 3D Slicer’s capabilities extend beyond that. For instance, it can integrate with hardware tracking systems for surgical navigation [31]. Moreover, the software continually evolves with the introduction of new extensions. Accessing 3D Slicer’s extensions is straightforward, through its extension “catalog.” Each extension is succinctly described and links to the extension’s website and source code are provided. Some examples of these diverse extensions include “Arduino Controller,” “Bone Reconstruction Planner,” “Breast Ultrasound Analysis,” “Kidney Stone Calculator,” “Matlab Bridge,” “Orthodontic Analysis,” “Perk Tutor,” “Slicer Elastix” (which brings Elastix’ functionalities to 3D Slicer), “Slicer Heart,” “Slicer IGT,” “Slicer Neuro,” “Slicer Radiomics,” “Slicer RT” (for radiation therapy). Through its extensions, 3D Slicer covers an exceptionally wide range of applications.

Besides 3D Slicer, MIPAV has a distinct advantage over the software assessed, due to the wide range of file types it supports, and a plethora of image manipulation utilities. For instance, users can easily combine 2D images into 3D images, or 3D to 4D images, crop images, resample them to share similar characteristics like orientation, resolution, and origin. But the software’s strength lies in its diverse collection of algorithms. For instance, image registration can be performed through a selection of 17 different methods. These registration methods can be further fine-tuned by its users. Finally, MIPAV expands its functionalities through more than twenty-five plugins. The plugins cover a wide range of applications, such as the plugins for “Axon Extraction,” “Brain Statistics,” “Brain Subcortical,” “CT abdomen,” “CT_MD,” “Dicom Reordering,” “Knees Femur segmentation,” “Neuron Segmentation,” “Stroke Segmentation,” “Synapse Detection,” “Worm Untwisting.” These plugins come with the basic installation of MIPAV, making their availability apparent to end-users. However, finding additional plugins developed and shared by other teams might prove challenging, as MIPAV does not have a centralized plugin catalog similar to what 3D Slicer offers.

#### Image Registration

Elastix emerged as the top-performing software for image registration. While it lacks a graphical user interface for manual image alignment, its automatic registration results proved to be the most accurate compared to other evaluated software. The registration functionality of Elastix was also highly convenient, as users could easily adjust registration parameters through a downloadable example file.

3D Slicer emerged as the second most efficient software for image registration. While manual registration is possible, users may need to follow tutorials as the process is not intuitively laid out. A wide range of registration parameters could be set for both 2D and 3D image registration cases.

ITK-SNAP secured the third position for image registration. Its main advantage over 3D Slicer and Elastix lies in the streamlined registration process and seamless verification of registration results through direct image fusion. However, ITK-SNAP’s weaknesses, compared to Elastix and 3D Slicer, were its limited parameter options for automatic registration setup. In addition, ITK-SNAP was less effective at registering 2D images than at registering 3D images.

MIPAV lacked options to configure the registration, and its registration results in both study cases were poorer than software which received higher rankings.

MedInria was often unresponsive when inserting markers during the manual registration. Additionally, some runs of the automatic registration led to application crashes. Nevertheless, when the software functioned properly, MedInria successfully aligned the images in both study cases.

MITK, Seg3D, and MeVisLab’s registration functionality encountered difficulties when working with 2D images. In its basic version, MITK only supports 3D-3D registration, lacking direct compatibility with 2D images. Similarly, Seg3D and MeVisLab failed to perform as expected with 2D images, despite their successful registration capabilities for 3D images. Seg3D is the most basic software for image registration; indeed, users would typically have to segment the target in the two images before being able to register the resulting masks.

#### Image Segmentation

The MITK workbench was the best software for image segmentation, primarily due to its straightforward and dependable manual and (semi-)automatic segmentation functionalities. In the 3D image test case, the manual segmentation of individual 2D slices was notably facilitated by the interpolation option. In the 2D image test case, users can only elect to employ the (semi-)automatic methods “threshold” and “threshold UL” options, while “Otsu,” “Picking,” and “GrowCut” could not be employed. Furthermore, MITK offered fewer post-processing tools for segmentation results compared to some other software solutions.

3D Slicer ranked second best software for image segmentation, as it offers a wide range of segmentation methods directly from its “Slicer core.” These methods include manual and (semi-)automatic approaches such as thresholding, region growing, and level tracing. The segmented results can undergo further processing (e.g., smoothing or removing small islands), and segmentation statistics (e.g., segmentation volume) can be calculated. The overall segmentation results were satisfactory for both image test cases, except when using the “region growing” segmentation method on the ultrasound image test case.

Seg3D secured the third position for image segmentation. Similar to several other software, Seg3D offers various segmentation methods, including manual approaches and (semi-)automatic options such as “Threshold” and “Otsu Threshold.” The “Otsu Threshold” tool demonstrated superior segmentation results compared to similar threshold-based tools in other software. To ease the manual segmentation process, users have access to the convenient “Speedline Tool.” Finally, Seg3D provides filters for post-processing segmentation results (e.g., dilate and erode functions).

ITK-SNAP offers intuitive and user-friendly manual and semi-automatic segmentation methods. These methods were quite dependable when applied to the 3D image test case. However, the (semi-)automatic methods performed poorly on the ultrasound image test case.

MIPAV’s manual and (semi-)automatic segmentation methods are supplemented by additional tools, such as for computing the Hausdorff distance between two segmentations or interpolating segmentations between slices of a 3D image. While the software demonstrated robustness when handling various types of images, it had subpar segmentation performance on the ultrasound image test case.

Segmentation using MeVisLab was not intuitive, but with the help of tutorials, it became manageable. When utilizing the (semi-)automatic methods, the resulting segmentation shapes did not match the real shape of the target organ (either a liver or a prostate); instead, a bias was exhibited towards producing brain-shaped segmentations.

Finally, MedInria’s segmentation process was frequently hindered by crashes. Moreover, the software offered only a few method options for segmenting images, limiting the user’s choices. The use of (semi-)automatic methods was challenging, which was exacerbated by the lack of documentation to guide users to employ these methods.

#### User-Friendliness

ITK-SNAP and MedInria were the most user-friendly software assessed. ITK-SNAP had the most intuitive user interface, ensuring that each action within the software flows smoothly. Users’ overall experience is seamless and efficient. MedInria also offers a streamlined user experience, although its interface was sometimes confusing. Indeed, numerous parameters are fitted into a single view, which can lead to users overlooking some that are required. This led to frustrating troubleshooting that was exacerbated by the use of small font sizes.

#### Documentation

Among all the assessed software in this paper, 3D Slicer has the most comprehensive documentation. In addition to be well-organized, it further includes examples and tutorials. The MITK Workbench also offers extensive documentation, although it is less engaging compared to 3D Slicer as it lacks examples and tutorials. Nonetheless, the MITK Workbench’s documentation maintains a uniform and coherent style.

#### Technical Support

Among the software options, 3D Slicer boasts the most active and helpful community. Users typically receive responses to their queries promptly, often within a few hours. Similarly, ITK-SNAP also maintains a supportive community where questions are addressed within hours. However, some questions remain unanswered occasionally. MITK also benefits from an actively supportive community, with users receiving responses to their questions within a few hours. However, fewer questions were asked compared to 3D Slicer and ITK-SNAP.

### A Closer Look at Individual Software

To help research teams with selecting a software according to the criteria that are important to them, detailed results for each software are presented in the following subsections.

#### 3D Slicer

Also known as “Slicer,” 3D Slicer is a popular open-source software in the CAI research community; indeed it has been downloaded over 1.2 million times since its 2011 release [32]. The software is distributed through a BSD-style license agreement and offers a plethora of functionalities, that can be further extended through modules and extensions; the modules are plug-ins that are already packaged as part of the 3D Slicer distribution, while extensions are external plug-ins that are installed “on-demand.”

While the software’s functionalities are comprehensive, novice users may find it challenging to familiarize themselves with it. Indeed, the user interface (UI) is overflowing with options that are not laid out in an intuitive order. Nonetheless, short tool descriptions are displayed when a user hovers over that tool. Furthermore, tutorials are available through videos, PowerPoint slides, and documents.

**Fig. 2:**
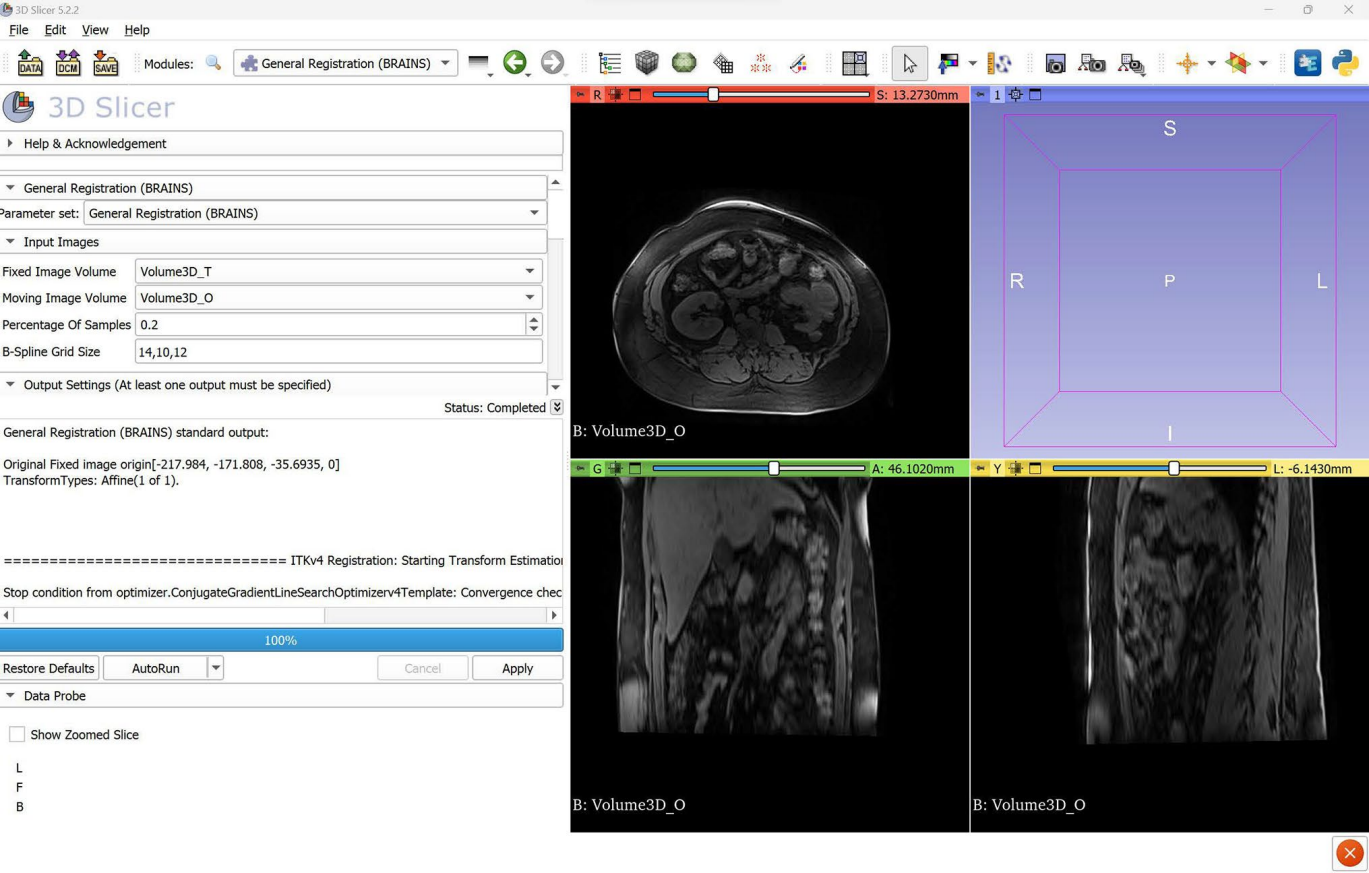
3D Slicer’s user interface. The registration functionality is being used

The software supports both image segmentation and registration (Fig. 2). A large number of methods are available to segment a region of interest. However, combining the different methods for segmentation, such as using the level tracing segmentation method, followed by manual segmentation for fine-tuning, could be challenging. The UI of 3D Slicer can present an obstacle when using the image registration functionality, as it is possible to overlook a required field, resulting in the error message “Status: Completed with errors.” This message is not easily noticeable in itself, further exacerbating the issue.

Technical support is excellent, with frequent queries and answers on forums. The software has the most detailed documentation compared to other software, but the presentation of functionalities is not always coherent; for instance, some functionalities were introduced through video tutorials, while others relied on PowerPoint slides. The use of PowerPoint slides, that are meant to be presented, can be challenging to interpret. Overall, 3D Slicer is a powerful software as it offers a wide range of functionalities that can be further extended if needed, it is extensively tested by its large community, it provides extensive documentation to users, and it has strong community support. Although its UI is not intuitive, proficiency in the software comes over time with experience.

3D Slicer exhibits the slowest initial start-up time compared to all the other evaluated software. Although 3D Slicer can be resource-heavy, it was able to load a large image file without overloading the system’s resources. Finally, multi-tasking is possible, but its RAM usage was the bottleneck.

#### Elastix

Elastix is open-source software designed solely to perform image registration. It has been distributed using the Apache 2.0 license since version 4.8. Elastix offers a fast and convenient way to quickly configure, test, and compare different registration strategies.

Elastix does not come with a graphical UI, and users typically resort to scripting to perform image registration. Users who are comfortable with typing a simple command in a terminal will appreciate Elastix’s straightforward approach to performing image registration, which avoids the confusing and click-heavy UIs of other software.

**Fig. 3:**
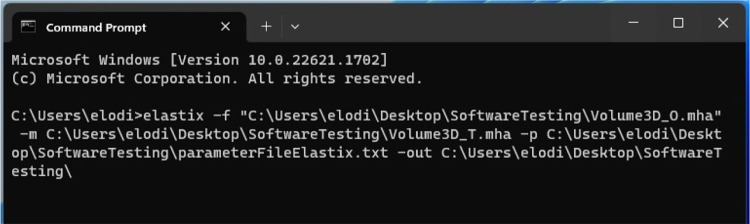
Command line for using Elastix’s image registration functionality

To initiate a registration task in Elastix, users only need to input a command in their terminal. Information to provide consists of the paths for the fixed (-f) and moving (-m) images to be registered, the text file (.txt) containing the registration parameters (-p) to be applied, and the destination folder (-out) for the registration results (Fig. 3). Visual verification of the registration results is not supported by this software, since it does not include a graphical UI. The users would therefore resort to other software, such as ITK-SNAP, to verify the registration results.

The Elastix documentation is comprehensive, although some of the links are outdated and inaccessible. For technical assistance, users can turn to a Google group, which typically receives a few messages per month. This communication method allows users to interact with both one another and the development team.

Given that Elastix does not have a graphical UI, the evaluations of the load time and stress load were not applicable. Nevertheless, its multi-tasking performance could be assessed. Elastix outperformed all other software in this regard. Indeed, although its CPU usage was slightly higher than that of ITK-SNAP, Elastix excelled at its low RAM consumption.

#### ITK-SNAP

**Fig. 4:**
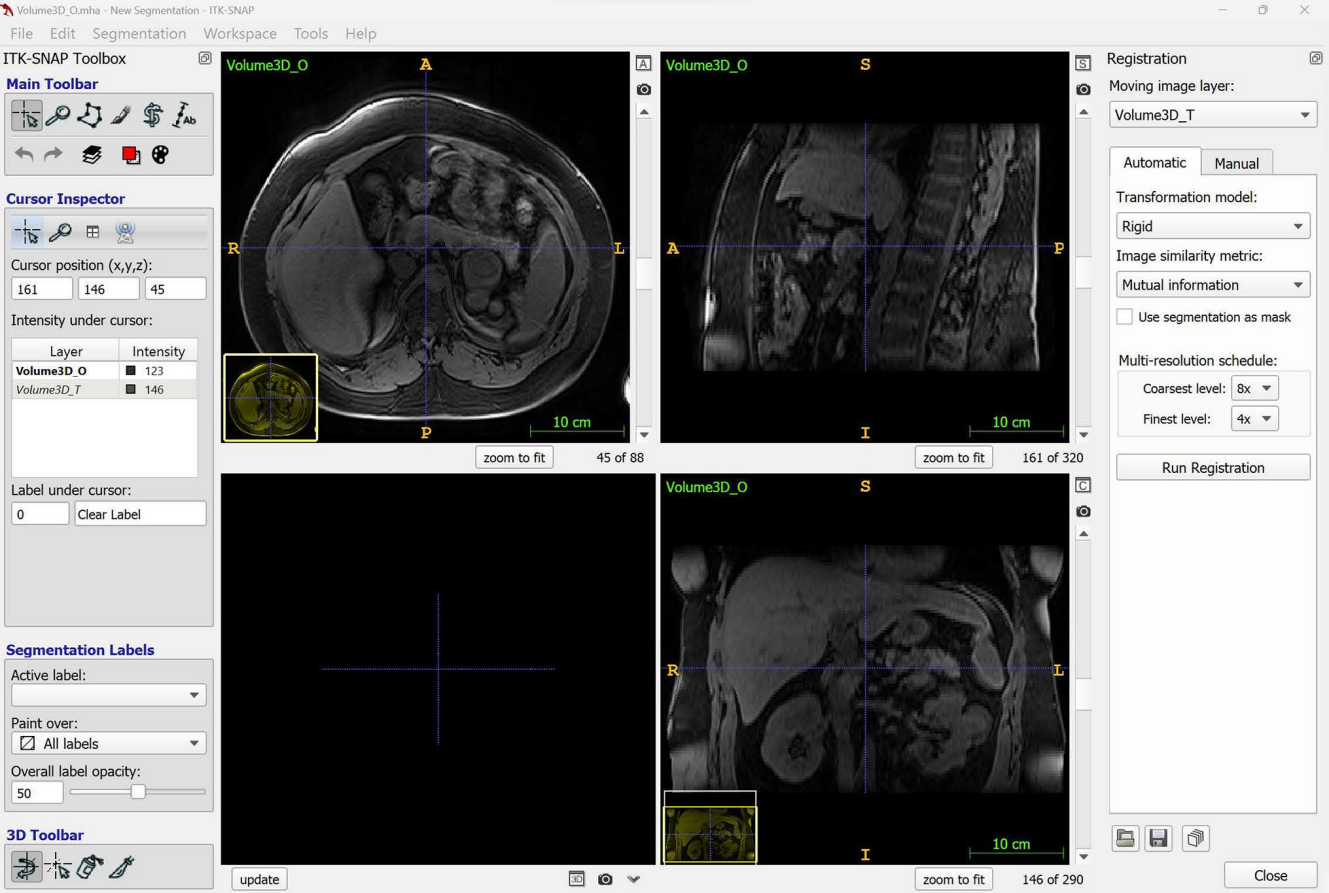
ITK-SNAP’s user interface. The registration tool is being used

Frequently referred to as “SNAP,” ITK-SNAP is another prevalent software used by CAI researchers; it is open-source and distributed under the GNU General Public License. ITK-SNAP is intended to be primarily a medical image visualization and segmentation software; nonetheless, it also allows users to perform image registration manually or using automatic methods (Fig. 4). End-users cannot develop plugins to extend its functionalities. The software’s interface is intuitive, presenting information coherently and with a well-designed layout that is not cluttered. Importing medical images is effortless, using either the file explorer or the drag-and-drop feature. There are also several visualization tools included, such as one to adjust the contrast of the images automatically and another one to adjust it manually. The segmentation and registration functionalities—both manual and (semi-)automatic—are overall dependable and intuitive to use.

While ITK-SNAP’s documentation is not as extensive as that of 3D Slicer, the information is well-organized and consistent in format. However, the documentation is mainly under the tab for ITK-SNAP’s versions 2.x, and its content has not been updated for ITK-SNAP’s versions 3.x. In addition, a few PDFs and a few tutorial videos on YouTube are available. Finally, there are three active ‘Google Groups’ forums available: one is for major announcements only, another is a discussion group for ITK-SNAP users, and the last one is a discussion group for ITK-SNAP developers.

ITK-SNAP is a lightweight software with the fastest initial start-up time. It is characterized by low RAM usage and utilizes both the GPU and CPU to process tasks. Additionally, it was able to load a large image file with ease, without causing the system to slow down.

#### MedInria

MedInria is an open-source software for medical image processing, that operates under a BSD-style licence. It was specifically built to be more reactive and intuitive than its counterparts [6]. While the UI is indeed more ergonomic than some popular software, it still presents some complexities.

**Fig. 5:**
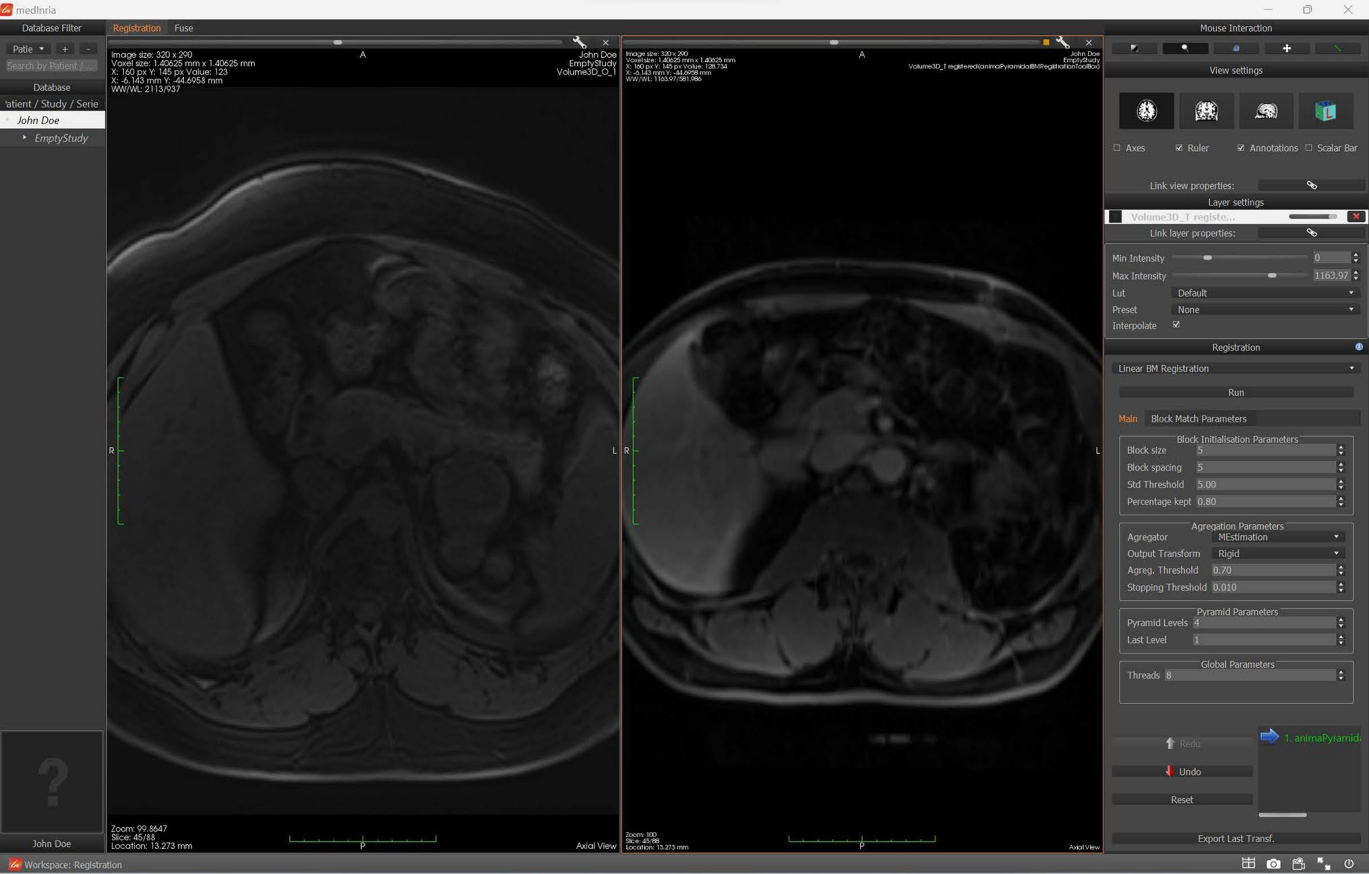
MedInria’s user interface: the image registration functionality is being used

The use of (semi-)automatic methods was challenging and, to the best of our knowledge, their documentation was missing. On the other hand, MedInria’s registration results were acceptable, when the software did not crash (Fig. 5). The registration results can be visualized from the “fuse” tab, where one can adjust the registered images’ opacities, to assess how well they were aligned.

MedInria’s documentation was sometimes found to have limited information about its functionalities. In addition, the documentation was outdated at times. MedInria has a YouTube channel that contains a handful of videos that could prove useful to end-users. Should end-users encounter technical difficulties, they can reach out to the development team by email. While a forum is also available, only 11 users have contributed to it, and the most recent message was posted 4 years ago, at the time of writing.

MedInria exhibited average start-up time, and high levels of RAM, CPU, and GPU usage when loading a large image file. While its performance score was average for this criterion, the only software programs that performed worse than MedInria were those which simply failed to load the large image. Finally, MedInria’s performance with respect to the multi-tasking criteria was below average, making it susceptible to crashes.

#### MeVisLab

MeVisLab is a software for medical image processing that differs from the other reviewed software, as it resorts to a graphical programming interface. Indeed, users should drag-and-drop, then interconnect, module blocks to create a custom image processing workflow. It is not open-source and offers several licensing options, including a free option for non-commercial institutions.

**Fig. 6:**
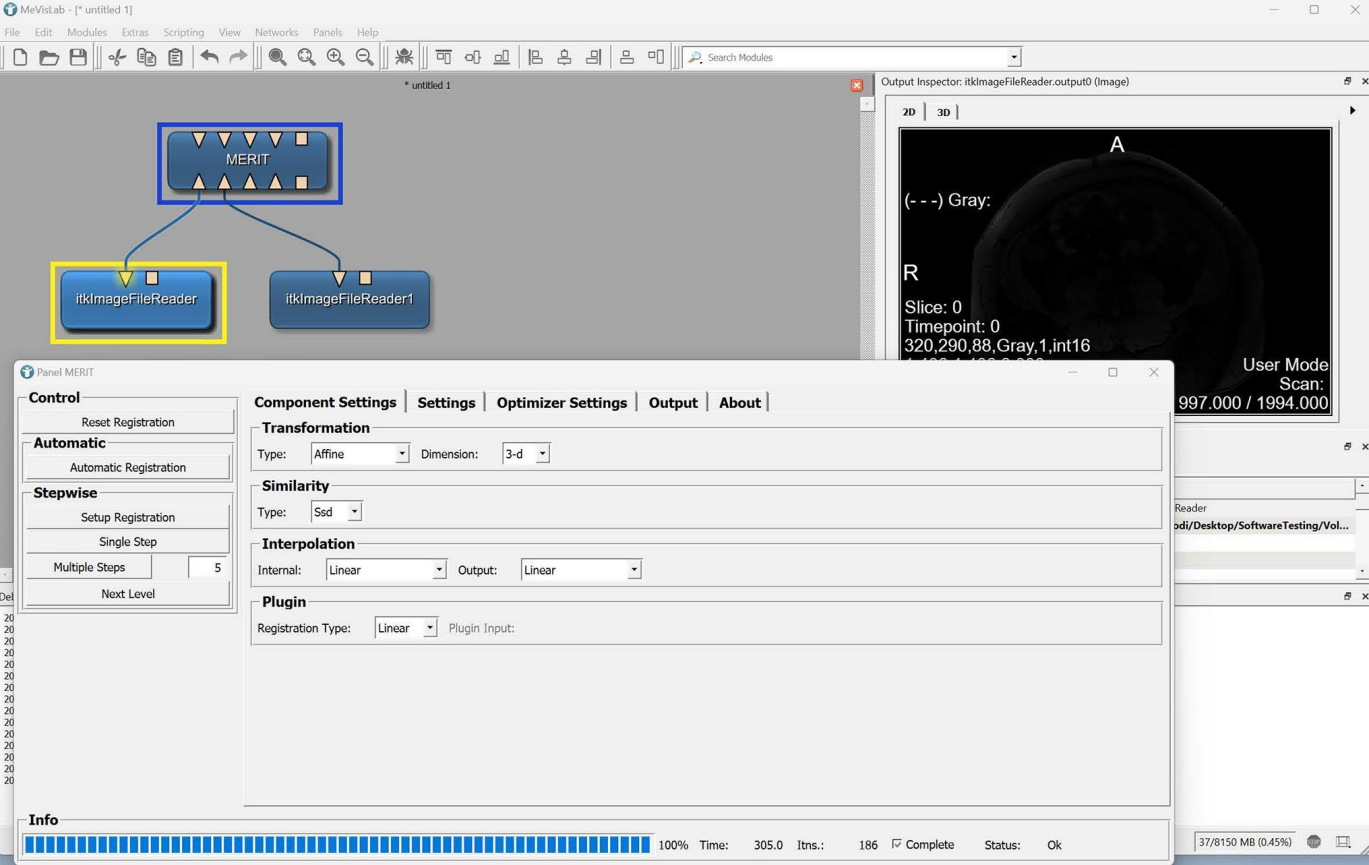
MeVisLab’s user interface: the image registration functionality is being used

In MeVisLab, users would typically begin by placing a module block that imports medical images; then a user would proceed to place other module blocks on the graphical programming interface and interconnect these blocks to create a custom image processing workflow. An example of such a module block would be ‘MERIT’ which performs image registration (Fig. 6).

A tutorial is provided; however, the tutorial relies on .tif images, which is unfortunate because medical images are frequently available in formats that contain metadata, such as DICOM. Indeed, the module block that is used to import .tif images is different from the module block that is used to import images with metadata. This can hinder the learning process of novice users who wants to assess whether this software is the right fit for their research needs. We struggled with the non-intuitive UI and found the documentation difficult to follow.

A forum is accessible for end-users. At the time of writing, the most recent message was posted approximately 2 months ago.

MeVisLab took a relatively long time to start up. It also failed to load the large image file and crashed in the process. Its multi-tasking performance was slightly above-average, mainly thanks to its low RAM usage.

#### MIPAV

**Fig. 7:**
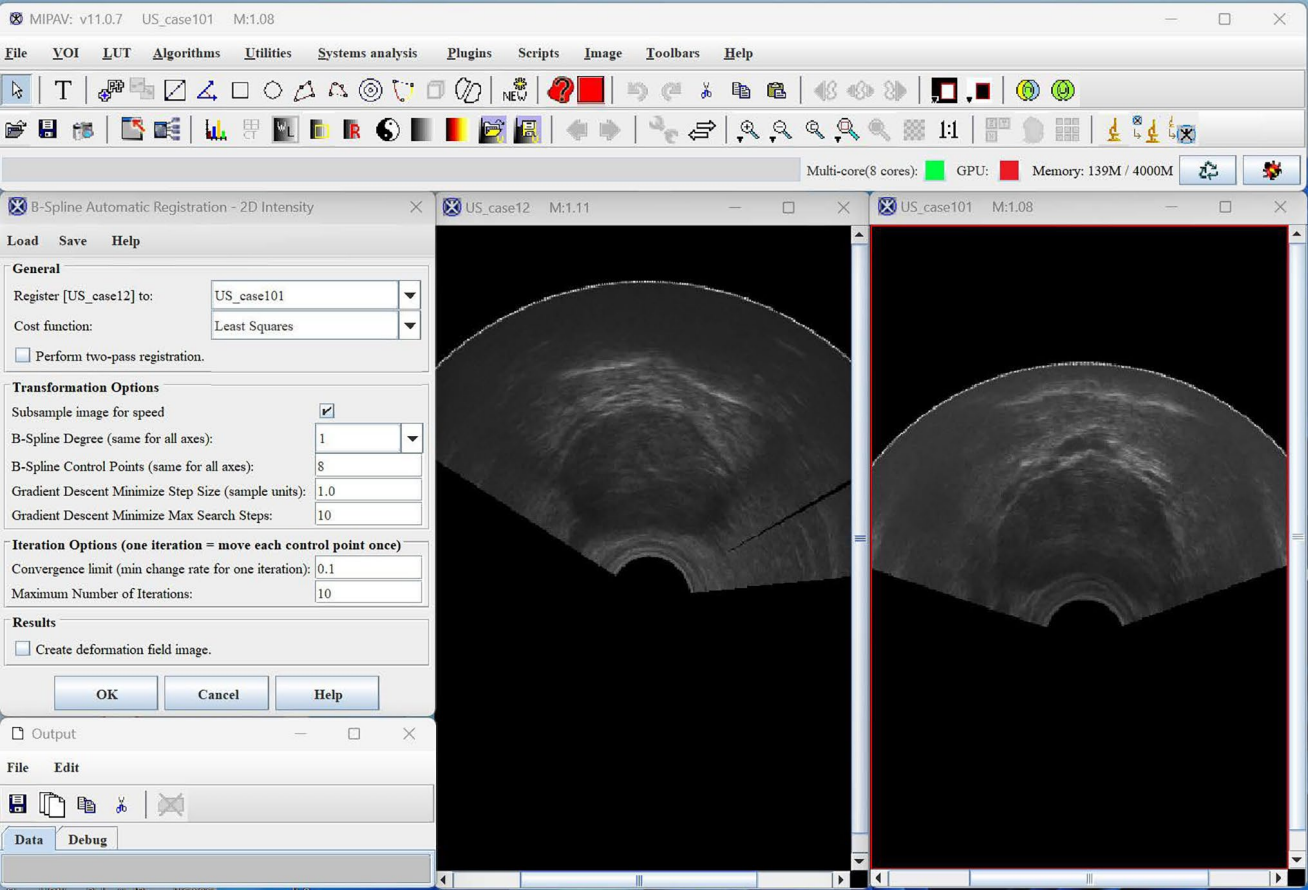
MIPAV’s user interface: the image registration functionality is being used

MIPAV, which takes its name after ‘Medical Image Processing, Analysis, and Visualization’, is another software that supports research endeavors in CAI. It is distributed under a modified BSD license. End-users can write MIPAV plugins to extend the software’s functionalities. MIPAV’s UI was neither intuitive nor user-friendly. Instead of being made of a single window, the UI is composed of multiple windows. For instance, as depicted in Fig. 7, five windows are opened when performing image registration.

The segmentation functionality includes several built-in tools, such as one to compute the Hausdorff distance between two segmentations, or one to interpolate segmentations between slices of a 3D image. Image registration is also supported by the software, with several metrics, transforms, and interpolation options available. Although the transformed images are presented to the user, it is unclear whether the actual numerical transform results can be extracted. Unfortunately, even if these functionalities work as intended, they are hindered by the UI of this software.

Documentation is available, but it is from 2008. Furthermore, to the best of our knowledge, there are no forums or ways to contact the development team for support.

MIPAV had a relatively short start-up time. In spite of that, it failed to load the large image file and crashed in the process. Ultimately, its multi-tasking performance was average, with RAM usage being the bottleneck.

#### MITK Workbench

The Medical Imaging Interaction Toolkit, better known as MITK, is a toolkit for the creation of CAI software. It is open-source, distributed under a BSD-style licence. MITK was strengthened through the addition of the MITK “Workbench.” Indeed, the MITK Workbench is a generic software that was created to provide basic functionalities in a graphical UI. The MITK Workbench can be extended using already-made plugins, and, just like 3D Slicer, end-users can create their own when the project requires advanced functionalities.

Uploading images is straightforward, and the interface is, overall, thoughtfully structured. The “View Navigator” tab gives swift access to all the available tools, such as general tools, as well as those for image registration and segmentation.

Manual and (semi-)automatic segmentation functionalities were fairly straightforward to use. However, it was sometimes noted that the segmentation algorithms stopped responding. This inconsistency lessens the program’s dependability because it repeatedly interrupts operations and fails to store users’ progress.

**Fig. 8:**
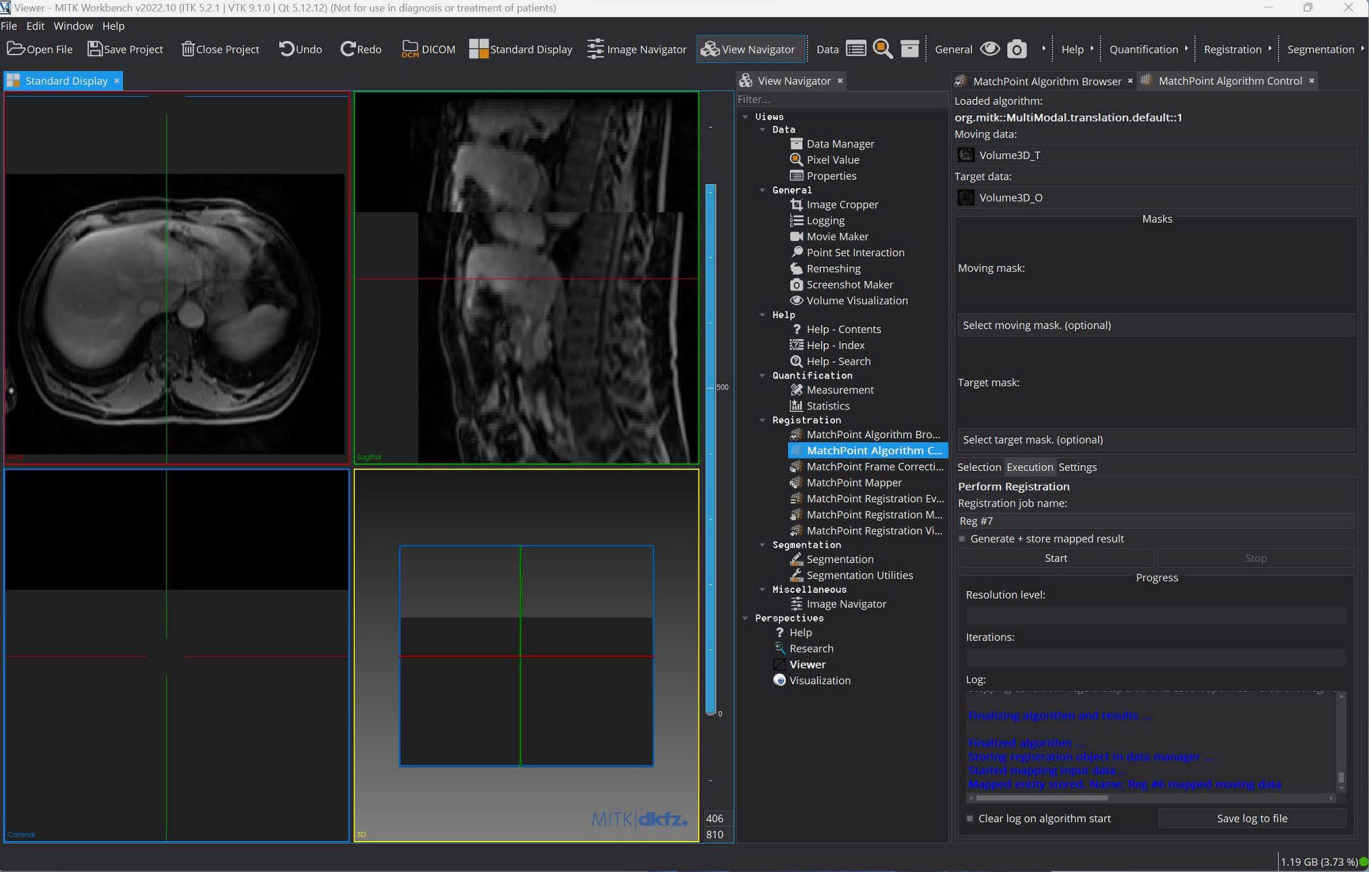
MITK Workbench’s user interface: the image registration functionality is being used

Novice users may find it difficult to perform automatic image registration due to the unclear process of loading the registration algorithm, and the lack of documentation or tutorial about it. To load the desired algorithm, users must first go to the “MatchPoint Algorithm Browser” tab and highlight the algorithm name. Then, they need to change tab, to the “MatchPoint Algorithm Control”; there, they need to navigate to the “Selection” tab (Fig. 8, approximately at the center-right of the window) and click on the “Load selected algorithm” button located in the middle of the UI. Setting the remaining registration parameters is more straightforward.

MITK’s documentation is comprehensive and organized. Furthermore, users have the option to email their questions to a mailing list. The email thread is posted online as it may be helpful to other users in the future.

Although the MITK Workbench had an average initial start-up time, it was one of the software that consumed the most resources during image registration: it had the highest CPU usage and it was among the software programs with the heaviest RAM usage.

#### Seg3D

Seg3D is an open-source software developed for image segmentation, that is shared under a BSD-style license (more specifically the MIT license). Like 3D Slicer and other software, Seg3D’s functionalities can be extended to cater to researchers’ specific project needs. Seg3D is lightweight and has interactive tools, such as image processing filters.

One helpful feature of Seg3D, is its ability to streamline the segmentation process. For instance, when segmenting the region of interest from a 3D image using the polygon tool, Seg3D remembers the its shape from the previous slice and can apply it to the subsequent slices.

**Fig. 9:**
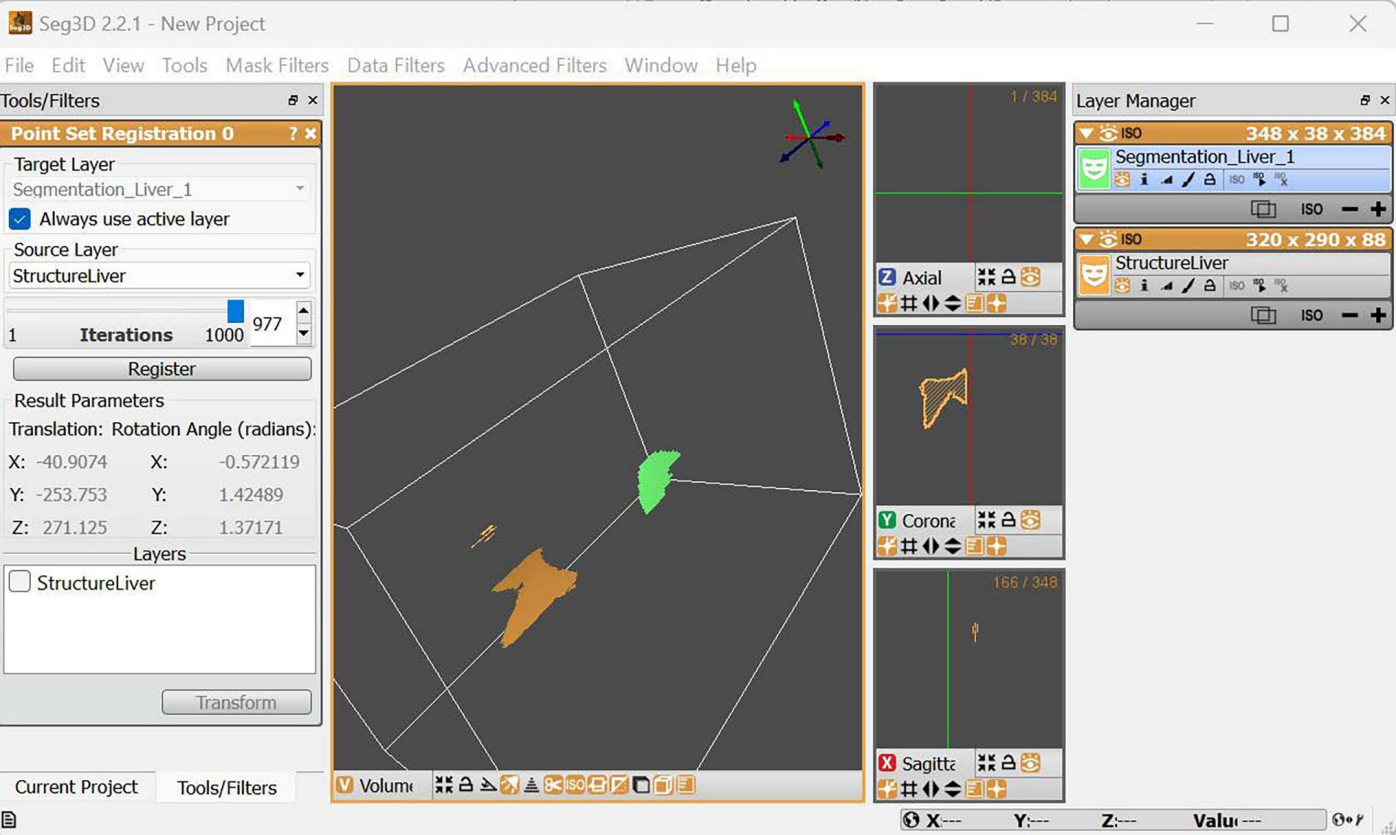
Seg3D’s user interface. Registration can be achieved by aligning two segmentations

Although this software is principally intended for image segmentation, image registration is, in a way, enabled. Indeed, image registration can be achieved by segmenting the target in two images and registering the resulting masks (Fig. 9 ).

Seg3D’s documentation can be confusing, but users can benefit from video tutorials. These video tutorials were particularly useful in getting accustomed to the software, given that the UI is not intuitive. Finally, users can communicate with the development team through a mailing list; it is worth noting, however, that the most recent visible email sent through the mailing list was about a year and a half ago, in October 2022.

Seg3D is a light application that loads quickly (the second-fastest). However, it failed to load the large image file and crashed in the process. Because image registration in Seg3D requires pre-processing (segmenting) the two images, and then registering the resulting masks, the registration algorithm was computationally less complex. In turn, comparing and including the results of the multi-tasking test would have been unfair.

## Discussion

### Interpretation and Limitations

Overall, ITK-SNAP and 3D Slicer were attributed the highest rankings. They could frequently complement each other, as ITK-SNAP is lightweight and is convenient for fundamental tasks, such as image visualization, while 3D Slicer has a larger and customizable range of functionalities. However, this study did not intend to provide a conclusion on the “best” software. In fact, each evaluated software was observed to have its own strengths and weaknesses. Assessments of the load time, stress load, and multi-tasking are more geared towards system administrators and IT infrastructure personnel as they are evaluating the required resource needed to support the software’s demands. Functionality assessments (visualization, registration, segmentation) are more geared toward actual end-users.

The purpose of evaluating the effectiveness of basic functionalities was to determine the usability, reliability, and amount of manual interaction required with the software. Due to the wide range of functionalities provided by the assessed software, we focused solely on assessing the segmentation and registration functionalities offered. The number of methods or parameters available for these functionalities did not influence the rankings given to this evaluation criteria. These were instead captured in the evaluation of the ’range of the functionalities’.

The evaluation of user-friendliness had some degree of subjectivity. Indeed, a UI that is intuitive to one user could be confusing to another, and luck may play a role in figuring out certain features. For instance, during the evaluation of the registration functionality of the MITK Workbench, loading the registration algorithm was reported to be challenging. To load the desired algorithm, the user had to go through different tabs, which were seemingly not connected. A novice user who stumbled upon the solution may find the process straightforward, while a user who spent days before discovering the solution will have a negative opinion of the UI design. A second example is the perception of Elastix’s user-friendliness: some novice users found it the most user-friendly, while others did not share the same sentiment because they were uncomfortable with typing a command in a terminal. User-friendliness results may differ based on individual preferences and past experiences.

The results of the multi-tasking tests should be interpreted with caution. Indeed, this test consisted in performing the automatic registration of two 3D images. However, it was not always possible to use the same registration parameters across all software. In the case of Seg3D, we deemed the registration functionality to be substantially different from that of the other software, and, consequently, the evaluation results were withheld.

Finally, we initially intended to conduct stress load tests by loading numerous 2D images, which is a more realistic scenario for researchers. However, certain software did not allow the simultaneous loading of multiple files. To ensure a fair comparison between the software, a synthesized large image was created. In turn, heavy workloads were simulated to put significant stress on the software.

### Excluded Software

Several selection criteria were utilized to narrow down the number of software to review. This was necessary because the CAI research community has a vast suite of software resources available.

The software “Analyze,” specifically designed for medical image processing and used in numerous research projects [33, 34], was not considered as it is not free of charge. A free version can be used for up to four months, and then the license has to be purchased and renewed on a yearly basis.

JULIUS [15] was excluded as it no longer appears to be shared online. Indeed, one criterion was that the software should be available online and be easily downloadable, without the need for administrative approval. If the software still exists, it could have a restricted availability and be distributed to a limited audience. Requesting to use it can be seen as inconvenient and complex, as it adds an extra layer of complexity and inconvenience for users. It may involve submitting requests, providing justifications, and waiting for responses, which can be time-consuming and discourage users from pursuing the software.

BrainVISA [9] is widely used in neuroscience research. However, it is not cross-platform as it operates on Linux; research investigators would be required to install other components, such as VirtualBox, if they have a Windows or macOS computer. Similarly, Osirix [2], a popular software for medical image processing, is available exclusively on macOS.

Several software programs were found to be infrequently maintained. For instance, the last release of GIMIAS [7] was in 2016, and, its official website (gimias.org) is no longer accessible. Similarly, AIBENCH [10] and OpenMAF [35] have been developed in the past, but have not been maintained for years. These are merely a few examples. While several large software programs encourage community involvement, many research groups still tend to develop their own software tailored to their specific requirements.

General-purpose software were excluded. In turn, ImageJ [36] (and its derivatives) and Paraview [37] were excluded, as they were developed for the broader scientific community rather than being specifically tailored to CAI initiatives.

There is a plethora of medical image viewers available; these were categorized as tools due to their specific purpose and limited range of capabilities. Typically, these viewers are principally designed to display images and to enable basic operations, such as annotations or measurements. VolView [38] is a popular image viewer, which does not require any installation since it is web-based. Another viewer is the Philips DICOM Viewer [39]. The software gives users the ability to measure distances, angles, and other characteristics on medical images as well as add annotations to the images.

Libraries, such as ITK [40] and VTK [41], were excluded even though they are among the most valuable resources for the CAI community. Indeed, the selected software had to be accessible to end-users without extensive programming experience.

## Conclusion

This review evaluated popular, freely distributed software specifically developed for the CAI research community. A total of eight software was identified: 3D Slicer, Elastix, ITK-SNAP, MedInria, MeVisLab, MIPAV, the MITK Workbench, and Seg3D. No software received the highest ranking in all the evaluation criteria. Due to the distinctive functionalities and performance of each software, it is impossible to put forward one specific software without, first, considering the requirements of a research project. Overall, ITK-SNAP and 3D Slicer emerged with the highest rankings. 3D Slicer and ITK-SNAP could complement each other, as the first one offers a wide range of functionalities that can be further extended, while the latter is lightweight and convenient for fundamental tasks, such as image visualization. The presented results also provide valuable insights to the software development teams, as they highlight areas where their software can be improved.
